# Supplementary material for: Comparing sources of mobility for modelling the epidemic spread of Zika virus in Colombia
Source: PLoS Negl Trop Dis. 2022 Jul 20;16(7):e0010565. doi: 10.1371/journal.pntd.0010565 (PMC9299334; doi:10.1371/journal.pntd.0010565)
Supplement: Attachment — Submitted filename: PNTD2022 - rebuttal letter.pdf [file pntd.0010565.s003.pdf]

Dear Editors,

Thank you for the opportunity to revise and resubmit our manuscript titled “Comparing sources of mobility for modelling the epidemic spread of Zika virus in Colombia” for consideration as a *PLOS Neglected Tropical Diseases* research article.

We are delighted to learn that the reviewers found our contribution interesting and worthy of publication with minimal revision. We carefully considered all suggestions, comments, and criticisms and revised the manuscript accordingly. We genuinely believe the reviewers' comments helped improve the manuscript considerably.

Although we provide in separate a detailed point-by-point response to the reviewers, we list here a summary of the main points addressed in the revised version of the manuscript:

- Following the remark of Reviewer #1, we included in our study an additional mobility network based on a version of the radiation network that we calibrated on the CDRs data instead of the census data. The census data indeed may underestimate the overall population movements in the country because of the retrospective data collection. Our findings show that the model estimates generated with this mobility network are comparatively similar to the ones obtained by the CRD-informed mobility network, with the advantage that in this case telecom providers would need to share only very aggregated information instead of the origin-destination matrix, thus adding an additional layer of data protection and privacy. We thank the reviewer for suggesting this possible extension to our work.
- Following the Editors' comments, we have improved the Discussion section by highlighting our key findings also in relation to previous studies in the literature.
- Following the comments by Reviewer #2, we have revised the entire manuscript to clarify those points that were not clearly explained.

We hope that the revised version of the manuscript addresses the reviewers' concerns and is suitable for publication in *PLOS Neglected Tropical Diseases*.

Thank you for your consideration of this manuscript.

Sincerely,
Daniela Perrotta on behalf of all co-authors

We would like to thank the reviewers for their valuable feedback on our manuscript that helped us to substantially strengthen our paper. Below we provide a point-by-point response (highlighted in blue) including a detailed description of all changes in the manuscript.

Editor(s) comments

This is a very well written manuscript with rigorous analyses. My main comments are for the discussion. The first paragraph could be strengthen with highlight the specifics of the results, what the key findings? And more discussion about how these specific results fit within the literature is needed.

We thank the editor for these comments. To improve the discussion section, we have now reformulated the first paragraph including a short summary of the key findings of our study. Furthermore we included additional references to previous studies in the literature. However, to the best of our knowledge, we are aware of only one previous study employing CDR-informed mobility into a spatial computational model in the context of ZIKV spread. Moreover, we checked and updated all references, converting preprints to published articles as appropriate.

Reviewer #1

This manuscript tackles two extremely relevant and timely topics at once. On the one hand, the authors present a computational model that is able to reproduce the empirical data on the outbreak of Zika virus in Colombia; most importantly on the other hand, the authors actually address the issue of which kind of mobility data sources are most suited to feed the model. Indeed, while very detailed data sources are becoming available, they are often used somehow "blindly" in models, without the demonstration that other types of data would not be as good.

Here, the authors build a metapopulation model and use 2 different data sources and 2 different mobility models to feed it: CDRs from mobile phone data, commuting data from a census, and the gravity and radiation models, adjusted to match the census patterns. The outcome of the model is compared to weekly time series of number of cases at the level of departments. The authors first compare the origin destination matrices of the 4 mobility data sources, highlighting both similarities and differences.They then show that the model is able in all cases to reproduce the epidemic peak, and yields a high correlation between the model and measured incidences at the country level.
When going into more details, by looking at the time series of the model's weekly incidence vs the observed weekly incidence in each department, they show that the best results are obtained with the CDR mobility data. Moreover, the performance of the CDR data is lower for departments where population and mobility fluxes are lower, while for the other data types performance is very heterogeneous.

Overall, the manuscript is well written, performs a timely and very relevant study, and certainly deserves publication.

We are truly glad to read these comments and thank the reviewer for their appreciation of our work.

I have two major points, on two very different aspects:

- it seems that data is not made available because it stems from mobile phone data. While I understand that the raw CDR data cannot be shared because it is proprietary, in the end the authors here use only very aggregated data, namely averaged flows at the scale of departments. I do not understand how such data cannot be shared, and I think it is crucial than an effort is made in this direction and the origin-destination matrices are made publicly available.

As we reported in the Data Availability Statement, the aggregated data used in this study can be made available after signing an NDA with the data-provider. While we understand and share the opinion of the reviewer about data openness, in this context we must follow the company policy that states that if a third party wants to access the data, even aggregated data, that can only be done after signing an NDA. The company feels it has the responsibility of being aware of how and for what applications the data set is being used for, and as such, has implemented an

NDA-policy. Ultimately, this is an additional layer required by the company to guarantee as much as possible the privacy of users.

- the gravity and radiation models are adjusted to the census data. However, as the authors note, the census data is from 2005. Therefore, it would seem more natural to adjust these models to the CDR data, to allow for a fairer comparison. Moreover, it would be then extra-interesting, if the gravity and/or radiation models were to perform well, as the resulting origin-destination matrices could then act as a substitute to data that cannot be shared.

We thank the reviewer for this important observation. We agree with the reviewer that the fact that the commuting census data is not recent but dates back to 2005 is a delicate point in our study. Nonetheless, it's the only official data source available thus far and our study aims to highlight the limited predictability in epidemic outbreaks in the absence of more refined and updated sources of mobility, as it is with CDRs data. While we want to maintain this narrative in our study, we believe that the reviewer raised an important point in terms of data sharing. Specifically, calibrating the radiation model on the CDR-informed mobility data would require only very aggregated information on the number of travelers in each location (i.e. the term T_i in Eq. 2 in the main text) which would serve to scale the flows based on the population density. In other words, this means that telecom providers would need to share only very aggregated data while preserving data protection and privacy. While this is true for the radiation model, it is not the case instead for the gravity model which would still need the raw origin-destination matrix from the CDRs data in order to fit the model.

Hence, following the reviewer's comment, we have now included in our analysis an additional mobility network based on an alternative version of the radiation model that we calibrated on the CDRs data instead of the census data. In the manuscript, we refer to this network as "hybrid radiation network". The flows in this new mobility network are proportionally higher than in the radiation network calibrated on the census data. This is due to the fact that the total outflows in the CDRs data are much higher than in the census data. Nonetheless, the flows in the two radiation networks are in good agreement with a Spearman correlation coefficient of 0.96. The epidemic results are comparatively similar to the ones obtained with the CDR-informed mobility network. This is evident in the new Figures 5,6,7 in the results section of the revised version of the manuscript. Our findings show that the model estimates generated by the CDR-informed mobility perform better in predicting the local outbreaks than the other mobility networks. This suggests that CDRs data, in any form, that is aggregated information on the total outflows or aggregated origin-destination matrices, better captures the mobility and mixing patterns relevant to predict the local spread of ZIKV infections.

To include this new analysis in our manuscript, we changed and included new sections both in the main text and in the Supplementary Materials, which we detail here below.

In the Methods, we included a new paragraph in the subsection "Synthetic mobility networks" to describe the additional mobility network calibrated on the CDRs data. This reads as follows (Lines 159-171 in the revised version of the manuscript):

“As a sensitivity analysis, since the most recent census data dates back to 2005, we create an alternative radiation network where T_i is calibrated on the CDR-informed mobility data instead of the census data. This requires only highly aggregated information about the total outflows in each department that serve as rescaling factor to the flows based on the population density. Hence, the goal here is to assess whether very aggregated information obtained from the CDRs, in contrast to the full dataset, may be sufficient to improve the quality of our epidemic modelling estimates. In the following, we will refer to this network as hybrid radiation network and denote it as $w_{ij}^{R,\ CDR}$. A comparison between the two radiation networks is provided in Section 4 in S1 Appendix, while we report the results of the epidemic model in the remainder of the main text. Note that a similar calibration could be used also for the gravity model but without added value in terms of data sharing as we would still need the complete origin-destination matrix from CDRs to fit the model.”

In the Supplementary Materials we have included a new section where we compare the hybrid radiation network calibrated on the CDRs data with the radiation network calibrated on the census data. This is Section 4 in the revised version of the manuscript.

In the Results section, we amended the figures and the text to include results obtained with the model estimates generated by the hybrid radiation network.

In the Discussion section, we addressed the advantages of the hybrid radiation network in terms of data sharing as telecom providers would need to share only highly aggregated information instead of the origin-destination that might sometimes be problematic in terms of data protection.

Reviewer #2

The manuscript "Comparing sources of mobility for modeling the epidemic spread of Zika virus in Colombia" provides a comparison of different mobility metrics including CDR data to model the spread of Zika across departments in Colombia. The paper is well written and provides a useful comparison of the different mobility patterns and how these impact model estimates. Below are some comments to address:

Major comments

1. Line 78: I see references for a few papers using mobility data for dengue among others – Are there other papers on ZIKA that include CDR mobility? Worth including if so

We thank the reviewer for this comment. To the best of our knowledge, we are aware of only one previous study employing CDR-informed mobility into a spatial computational model in the context of ZIKV spread. We referred to this study in the Discussion section of the original manuscript (Lines 450-452):

> “A recent study on the 2015-2016 Zika virus epidemic in Colombia showed that an ensemble modelling approach integrating multiple data sources for human mobility, including CDR-derived mobility, is prominent to forecast an emerging infectious disease like Zika [46].”

To address the reviewer’s comment, we have now included this reference also in the Introduction section of the revised version of the manuscript (Lines 39-43) which reads as follows:

> “The availability of aggregated CDR-derived mobility has impacted several research fields [16], with significant applications to the spatial modelling of many infectious diseases, such as malaria [17, 18], dengue [19], cholera [20], rubella [21], Ebola [22, 23], ZIKV [24], and COVID-19 [25, 26, 27, 28, 29].”

2. Line 183: Could the authors expand on how the flows were rescaled by the population for the Census Network?

We thank the reviewer for this comment. Since the commuting data refers to the 2005 Colombian census, here we employed a standard weighting approach to account for potential changes in population that occurred during this time period. For this, we simply rescaled commuting flows based on the population ratio between the 2005 Colombian census and the 2015 population estimates.

To further clarify this point, we have now expanded the description in the revised version of the manuscript (Lines 132-135) which reads as follows:

> “We aggregate flows spatially into departments and rescale them to reflect the 2015 population estimates. To this aim, we reweight flows on the population ratio between the 2005 Colombian census and the 2015 population estimates, in order to account for the population changes that occurred over this period.”

3. Line 303: in each of the panels in Figure 4, there’s a noticeable outlier – did the authors evaluate the change in the correlation estimate with the removal of the outlier, or how much it might be impacting those trends?

We thank the reviewer for this observation. If we understand correctly, the outlier mentioned corresponds to the highest flow of individuals that occurs between the Capital District Bogotà and the nearest department of Cundinamarca, as shown in Figure 3. While this is an outlier in the distribution of flows, it does not deviate substantially from the regression line when compared to the census network in Figure 4. The correlations in fact are not affected, up to the third decimal place, for all networks.

4. Line 322: How does the model’s estimated peak incidence compare to what was officially reported?

We thank the reviewer for this question. We addressed this point in the original manuscript (Lines 316-323). As typically happens for asymptomatic or mild diseases, the model estimated ZIKV incidence is much larger than the observed one. This is evident in Figure 5 where, for the sake of comparability, we plot the curves on different y-axes, i.e., the official surveillance data on the right y-axis against the model estimates on the left y-axis. There’s indeed two orders of magnitude of difference at the peak.

To further clarify this point, we revised the text as follows (Lines 310-318):

> “In Fig 5, we show the estimated weekly incidence of ZIKV infections (per 100,000 population) in comparison with the official surveillance data reported by Colombia's National Institute of Health (INS). For ease of comparison, the latter is showed on a different y-axis matching the peak of the model estimates of the CDR-informed network. This is because the model projects a much larger number of infections than those captured by the surveillance system, as expected for a typically asymptomatic or mild disease. In particular, based on the official surveillance data, the epidemic peak occurred in week 2016-05 with an incidence of approximately 10 cases per 100,000 population, which results in a case ascertainment rate of about 1% for the reporting system at the peak of the epidemic.”

5. Line 361: How does the model estimated total case compare to the observed?

We addressed this point in the original version of the manuscript. We estimated the reporting and detection rate by comparing the total number of cases in the model estimates vs. the observed data through a linear regression fit (Lines 355-357). This ranges between

0.51%±0.23% for the gravity network to 0.72%±0.32% for the CDR-informed network, confirming the two-orders-of-magnitude difference we estimated between our model estimates and observed surveillance data. Figure 9 in the Supplementary Material further shows this relationship by comparing the median values of the model estimates and the official surveillance data by department.

To better highlight this point, we have now amended the text accordingly in the revised version of the manuscript (Lines 354-359) which reads as follows:

> “To compare the total ZIKV cases projected in our model estimates against those observed in the official surveillance data, we estimate a reporting and detection rate through a linear regression fit (see top panel of Fig 11 in S1 Appendix). The estimated detection rate ranges between 0.51% ± 0.23% for the gravity network to 0.72% ± 0.32% for the CDR-informed network (all $p<0.05$), thus confirming for the detection and reporting system the ascertainment rate we estimated at the national level.”

Line 452: Are there seroprevalence estimates for Colombia or particular departments that can be compared with the estimates derived from the model?

As this is a delicate point in our study, we extensively described the epidemiological data in the first section of the Supplementary Material. Specifically, we reported the cumulative ZIKV incidence by department in Colombia in Figure 1 and Table 1 of the Supplementary Material. The number of laboratory-confirmed cases represents only 8% of the total reported cases, which makes it very difficult to use as ground truth for our study as data would be very sparse, especially the weekly times series. In our study, where we used the total number of reported cases, we already had to exclude a number of departments having less than 100 total ZIKV cases reported by the official surveillance data (Lines 330-332 of the original manuscript). Using the laboratory-confirmed cases would further hinder the comparison of our model estimates and therefore the assessment of the performance of the various mobility patterns considered in this study. Furthermore, previous studies showed that underreporting of ZIKV cases, due to for instance limited diagnostic capabilities, medically unattended cases, and asymptomatic infections, may have contributed significantly to underestimating the actual extent of the epidemic. We addressed this point among the limitations of our study in the Discussion section of the original manuscript (Lines 425-437). The weekly epidemiological reports from the Colombian National Institute of Health are often inconsistent or inadequate with numbers of cases varying significantly over time and comparatively low detection of laboratory-confirmed cases. This represents an additional challenge in our study as we use this dataset as a reference to assess the model performance in reproducing the ZIKV outbreak.

Minor comments:

1. Will the R code be made publicly available?

Data analysis and modelling performed in this study consists of several scripts and notebooks in different programming languages, from Python to C++ to Bash. Some of the developed code cannot work without the original CDR data. We will honor requests to share the original code that are respectful of the original CDR data sharing agreement.
